# Cohort Profile Update: The Finnish Gestational Diabetes (FinnGeDi) study

**DOI:** 10.1093/ije/dyag101

**Published:** 2026-07-09

**Authors:** Shilpa Lingaiah, Elina Keikkala, Sanna Mustaniemi, Pekka Ylöstalo, Tellervo Tervonen, Marja-Liisa Laitala, Hilkka Pernu, Eveliina Lammentausta, Mika Nevalainen, Jyri-Johan Paakki, Satu Männistö, Niina E Kaartinen, Marjaana Tikanmäki, Annukka Torkki, Fredrik Åberg, Anneli Pouta, Risto Kaaja, Johan G Eriksson, Hannele Laivuori, Mika Gissler, Tuija Männistö, Pirkko Pussinen, Terhi Ruuska-Loewald, Kari Anne I Evensen, Mikko Tulppo, Julia Jäkel, Eero Kajantie, Marja Vääräsmäki

**Affiliations:** Research Unit of Clinical Medicine, Medical Research Centre Oulu, Oulu University Hospital and University of Oulu, Oulu, Finland; Population Health Unit, Finnish Institute for Health and Welfare, Helsinki/Oulu, Finland; Research Unit of Clinical Medicine, Medical Research Centre Oulu, Oulu University Hospital and University of Oulu, Oulu, Finland; Population Health Unit, Finnish Institute for Health and Welfare, Helsinki/Oulu, Finland; Research Unit of Clinical Medicine, Medical Research Centre Oulu, Oulu University Hospital and University of Oulu, Oulu, Finland; Population Health Unit, Finnish Institute for Health and Welfare, Helsinki/Oulu, Finland; Research Unit of Population Health, Medical Research Centre Oulu, Oulu University Hospital and University of Oulu, Oulu, Finland; Research Unit of Population Health, Medical Research Centre Oulu, Oulu University Hospital and University of Oulu, Oulu, Finland; Research Unit of Population Health, Medical Research Centre Oulu, Oulu University Hospital and University of Oulu, Oulu, Finland; Research Unit of Population Health, Medical Research Centre Oulu, Oulu University Hospital and University of Oulu, Oulu, Finland; Research Unit of Health Sciences and Technology, Medical Research Centre Oulu, Oulu University Hospital and University of Oulu, Oulu, Finland; Research Unit of Health Sciences and Technology, Medical Research Centre Oulu, Oulu University Hospital and University of Oulu, Oulu, Finland; Research Unit of Health Sciences and Technology, Medical Research Centre Oulu, Oulu University Hospital and University of Oulu, Oulu, Finland; Department of Public Health, Finnish Institute for Health and Welfare, Helsinki, Finland; Department of Public Health, Finnish Institute for Health and Welfare, Helsinki, Finland; Research Unit of Clinical Medicine, Medical Research Centre Oulu, Oulu University Hospital and University of Oulu, Oulu, Finland; Population Health Unit, Finnish Institute for Health and Welfare, Helsinki/Oulu, Finland; Research Unit of Clinical Medicine, Medical Research Centre Oulu, Oulu University Hospital and University of Oulu, Oulu, Finland; Population Health Unit, Finnish Institute for Health and Welfare, Helsinki/Oulu, Finland; Transplantation and Liver Surgery, Helsinki University Hospital and University of Helsinki, Helsinki, Finland; Department of Government Services, Finnish Institute for Health and Welfare, Helsinki, Finland; Institute of Clinical Medicine, Internal Medicine, Turku University Hospital, University of Turku, Turku, Finland; Department of General Practice and Primary Health Care, Helsinki University Hospital, University of Helsinki, Helsinki, Finland; Folkhälsan Research Centre, Helsinki, Finland; Department of Obstetrics and Gynaecology, Human Potential Translational Research Programme, Yong Loo Lin School of Medicine, National University of Singapore, Singapore, Singapore; Medical and Clinical Genetics, University of Helsinki and Helsinki University Hospital, Helsinki, Finland; Institute for Molecular Medicine Finland, Helsinki Institute of Life Science, University of Helsinki, Helsinki, Finland; Department of Obstetrics and Gynaecology, Tampere University Hospital, The Wellbeing Services County of Pirkanmaa, Tampere, Finland; Center for Child, Adolescent, and Maternal Health Research, Faculty of Medicine and Health Technology, Tampere University, Tampere, Finland; Department of Data and Analytics, Finnish Institute for Health and Welfare, Helsinki, Finland; Research Centre for Child Psychiatry, University of Turku, Turku, Finland; Academic Primary Health Care Centre, Region Stockholm, Stockholm, Sweden; Department of Molecular Medicine and Surgery, Karolinska Institutet, Stockholm, Sweden; Clinical Chemistry, Institute of Clinical Medicine, University of Eastern Finland, Kuopio, Finland; Joint County Authority for ISLAB Laboratories, Kuopio, Finland; Translational Medicine Research Unit, University of Oulu, Oulu, Finland; Department of Oral and Maxillofacial Diseases, University of Helsinki, Helsinki, Finland; Institute of Dentistry, University of Eastern Finland, Kuopio, Finland; Research Unit of Clinical Medicine, Medical Research Centre Oulu, Oulu University Hospital and University of Oulu, Oulu, Finland; Department of Clinical and Molecular Medicine, Norwegian University of Science and Technology, Trondheim, Norway; Department of Rehabilitation Science and Health Technology, Oslo Metropolitan University, Oslo, Norway; Research Unit of Biomedicine and Internal Medicine, Medical Research Centre Oulu, University of Oulu and Oulu University Hospital, Oulu, Finland; Unit of Psychology, University of Oulu, Oulu, Finland; Department of Psychology, Health and Medical University Düsseldorf, Düsseldorf, Germany; Department of Psychology, University of Warwick, Coventry, United Kingdom; Research Unit of Clinical Medicine, Medical Research Centre Oulu, Oulu University Hospital and University of Oulu, Oulu, Finland; Population Health Unit, Finnish Institute for Health and Welfare, Helsinki/Oulu, Finland; Department of Clinical and Molecular Medicine, Norwegian University of Science and Technology, Trondheim, Norway; Children’s Hospital, University of Helsinki and Helsinki University Hospital, Helsinki, Finland; Research Unit of Clinical Medicine, Medical Research Centre Oulu, Oulu University Hospital and University of Oulu, Oulu, Finland; Population Health Unit, Finnish Institute for Health and Welfare, Helsinki/Oulu, Finland

**Keywords:** gestational diabetes, long-term follow-up, FinnGeDi cohort

Key Features

- The Finnish Gestational Diabetes (FinnGeDi) study was set up in 2009–12 to investigate various aspects of gestational diabetes (GDM) after the introduction of the Current Care Guidelines in Finland.
- The present 11- to 15-year follow-up provides opportunities for new research on the multidimensional understanding of health in women after a GDM pregnancy, including cardiometabolic and oral health, as well as psychosocial well-being.
- The follow-up study population consists of 213 women with a history of GDM and 204 control women aged 30–60 years (mean age 44.3 years) currently residing in the Oulu region of Finland.
- New measures include dietary data, oral health, accelerometer-measured physical activity, heart-rate variability, assessment of psychosocial well-being, imaging, and microbiome studies.
- For collaboration, please contact Professor Marja Vääräsmäki at marja.vaarasmaki@oulu.fi.

## The original cohort

The Finnish Gestational Diabetes (FinnGeDi) study was set up in 2009 to investigate various aspects of gestational diabetes (GDM) after the introduction of the Finnish Current Care Guidelines, in which comprehensive screening was recommended to replace the previous risk-factor-based screening for GDM [1]. Accordingly, all women, except those with a very low risk, were recommended to be screened for GDM by using a 75-g 2-hour oral glucose tolerance test (OGTT) between 24 and 28 weeks of pregnancy. For women at high risk, the first OGTT was recommended earlier, between 12 and 16 weeks, and repeated at 24–28 weeks if the initial result was normal.

As outlined in the original cohort profile paper, the FinnGeDi study consists of two arms: a case–control arm and a register-based arm [2]. The present follow-up study is based on the case–control arm, which included medical, obstetric, perinatal, and neonatal data in addition to information on family members and participants’ own medical histories, obtained through questionnaires, medical records, and the Finnish Medical Birth Register. Furthermore, blood samples were collected for DNA extraction and further analysis from pregnant women with and without GDM, as well as from their children and the children’s fathers. The original case–control cohort consisted of 1146 pregnant women with GDM and 1066 women without GDM, their children from the index pregnancy, and the children’s fathers. GDM diagnosis was based on an abnormal OGTT result during pregnancy. Based on the Finnish Current Care Guidelines, the cut-off values for plasma glucose were 5.3 mmol/L at baseline (fasting glucose), 10.0 mmol/L at 1 h, or 8.6 mmol/L at 2 h after glucose intake [1]. GDM diagnosis was made if one or more glucose concentrations exceeded the cut-off levels. Women with pre-pregnancy diabetes and multiple pregnancies were excluded from the study. Participants consented in writing to be contacted for follow-up studies.

The study aimed to identify the risk factors and clinical characteristics of GDM, as well as genetic and epigenetic biomarkers associated with GDM. The incidence, distribution, and consequences of GDM were to be assessed in different demographic groups and across generations.

## What is the reason for the new data collection?

One of the original aims of establishing the FinnGeDi cohort was to form a study cohort suitable for long-term follow-up by using both clinical assessments and national health registers. A key aim was to focus on the long-term consequences of GDM. For the present clinical follow-up, the data collection was conducted 11–15 years after the index pregnancy.

## What will be the new areas of research?

One of the novel areas of the follow-up is to study the prevalence of metabolic dysfunction-associated steatotic liver disease (MASLD), formerly known as non-alcoholic fatty liver disease (NAFLD), 11–15 years after GDM pregnancy. Liver biopsy remains the gold standard for diagnosing steatohepatitis. However, its use is limited due to invasive risks and high costs [3, 4]. Magnetic resonance imaging (MRI) is considered the non-invasive gold standard for lipid quantification in MASLD [5, 6]. In the present study, a diagnosis of MASLD is based on MRI and transient elastography. Also, scoring indices, including the Hepatic Steatosis Index, Fibrosis-4 Index (FIB-4), and NAFLD Fibrosis Score, are calculated, allowing comparisons between different non-invasive diagnostic methods used to assess MASLD.

Dietary habits and nutritional factors play a significant role in overall health, including oral health. Oral health is an integral part of general health but is often neglected and has been less studied in GDM follow-up studies. Furthermore, dietary habits, poor oral health, and chronic diseases are interrelated [7]. In the FinnGeDi follow-up study, combining dietary assessment with oral health examination provides new insights into the relationship between nutrition, oral health, and chronic-disease development. Additionally, exploring potential associations of dental caries and periodontitis with MASLD will enhance our understanding of the pathogenesis and progression of MASLD.

Other important focuses of the follow-up are the oral and the gut microbiomes. Altered microbiome or dysbiosis is linked to various systemic and metabolic diseases, including obesity and type 2 diabetes (T2D) [8]. Studies have reported that women with GDM present with altered gut microbiome [9]. However, it is unknown whether and how long these alterations persist after GDM pregnancy or whether they are associated with progression to T2D. Understanding the interplay between oral and gut microbiomes and their association with MASLD and T2D will provide novel insights into both risk and protective factors.

Depression and anxiety are linked to adverse health behaviours, such as unhealthy diet and reduced physical activity, which also affect quality of life [10, 11]. The associations of psychosocial stress, depressive symptoms, and anxiety with GDM have been less studied. Few studies have shown an association between GDM and the subsequent development of depression and anxiety, with women with GDM exhibiting a four-fold increased risk of postpartum depression [12]. Of note, the ‘Women’s Health Report’ by the World Health Organization in 2016 reported a higher incidence of mental health issues in reproductive age women, although the data for long-term mental health sequelae associated with GDM are lacking [13]. We study the psychological aspects by using standardized and validated questionnaires, which will help us understand the psychosocial well-being of women with previous GDM.

The study will also explore the role of physical activity. Studies in the general population have shown that moderate-to-vigorous physical activity is associated with a 25%–40% reduction in the risk of T2D [14]. However, there is a lack of studies examining the relationship between physical activity and the development of MASLD among women with prior GDM, and it is unclear whether specific types of physical activity are more effective in reducing this risk.

## Who is in the cohort?

**Figure 1:**
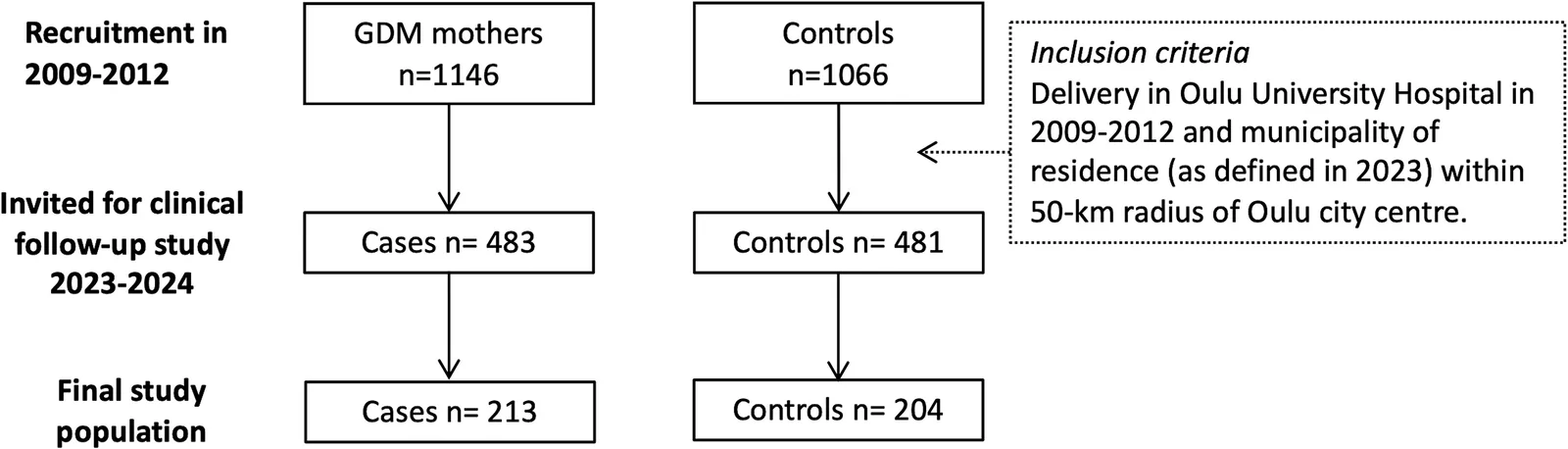
Flow chart of women in the clinical follow-up study.

The current clinical follow-up study, focusing on a subset of women from the original case–control cohort who had delivered at Oulu University Hospital and were residing within a 50-kilometre radius of the city centre of Oulu, Finland at the time of data collection, was carried out during March 2023–October 2024. The included cases had GDM, while the controls did not have GDM in the index pregnancy. The participants were contacted by an invitation letter after their addresses were retrieved from the population information system (Digital and Population Data Services Agency). Finally, 964 women (483 with a history of GDM and 481 controls) were invited in the same order as they were recruited into the original study. The exclusion criterion for participation in the follow-up study was ongoing pregnancy. Contraindications for MRI (e.g. metallic implants or pacemakers) prevented participation in the MRI examination. The final study population consisted of 213 women with prior GDM and 204 control women, with participation rates of 44.1% and 42.4% (*P *= .597), respectively (Fig. 1). The baseline characteristics of the participants and non-participants stratified by GDM status are shown in Table 1. Participants were slightly older, had lower pre-pregnancy body mass index (BMI), and were more likely to have higher education and less likely to smoke during pregnancy compared with non-participants. No differences were observed for parity, primiparity, or pharmacological treatment of GDM.

**Table 1:** Baseline characteristics of non-participants and participants

Parameter	Non-participants (*n* = 547)			Participants (*n* = 417)		
	GDM (*n* = 270)	Controls (*n* = 277)	*P* value	GDM (*n* = 213)	Controls (*n* = 204)	*P* value
Age at index delivery (years)	30.9 (5.4)	28.0 (4.7)	<.001	32.5 (5.1)	30.4 (5.1)	<.001
Pre-pregnancy BMI (kg/m^2^)	28.6 (6.1)	23.6 (4.2)	<.001	26.3 (5.2)	23.5 (3.6)	<.001
Parity	1 (0–2)	0 (0–2)	.204	1 (0–2)	1 (0–1.75)	.079
Primiparous	119 (44.1)	139 (50.2)	.153	79 (37.1)	94 (46.1)	.063
Education	249	247	.019	201	191	.971
Basic or less	28 (11.3)	16 (6.4)		6 (3.0)	6 (3.1)	
Secondary	115 (46.6)	135 (54.2)		78 (38.8)	72 (37.7)	
Lower-level tertiary	66 (26.7)	47 (18.9)		64 (31.8)	65 (34)	
Upper-level tertiary	38 (15.4)	51 (20.5)		53 (26.4)	48 (25.0)	
Smoking during pregnancy	55 (20.4)	63 (22.7)	.514	18 (8.5)	13 (6.4)	.459
Pharmacological treatment of GDM	54 (20.0)	–		31 (14.6)	–	

Data shown as mean (SD), median (interquartile range), or number (%). *P* values from *t*-test, Mann-Whitney test, or chi-square tests.

BMI, body mass index; GDM, gestational diabetes. –, data not applicable.

## What has been measured?

**Figure 2:**
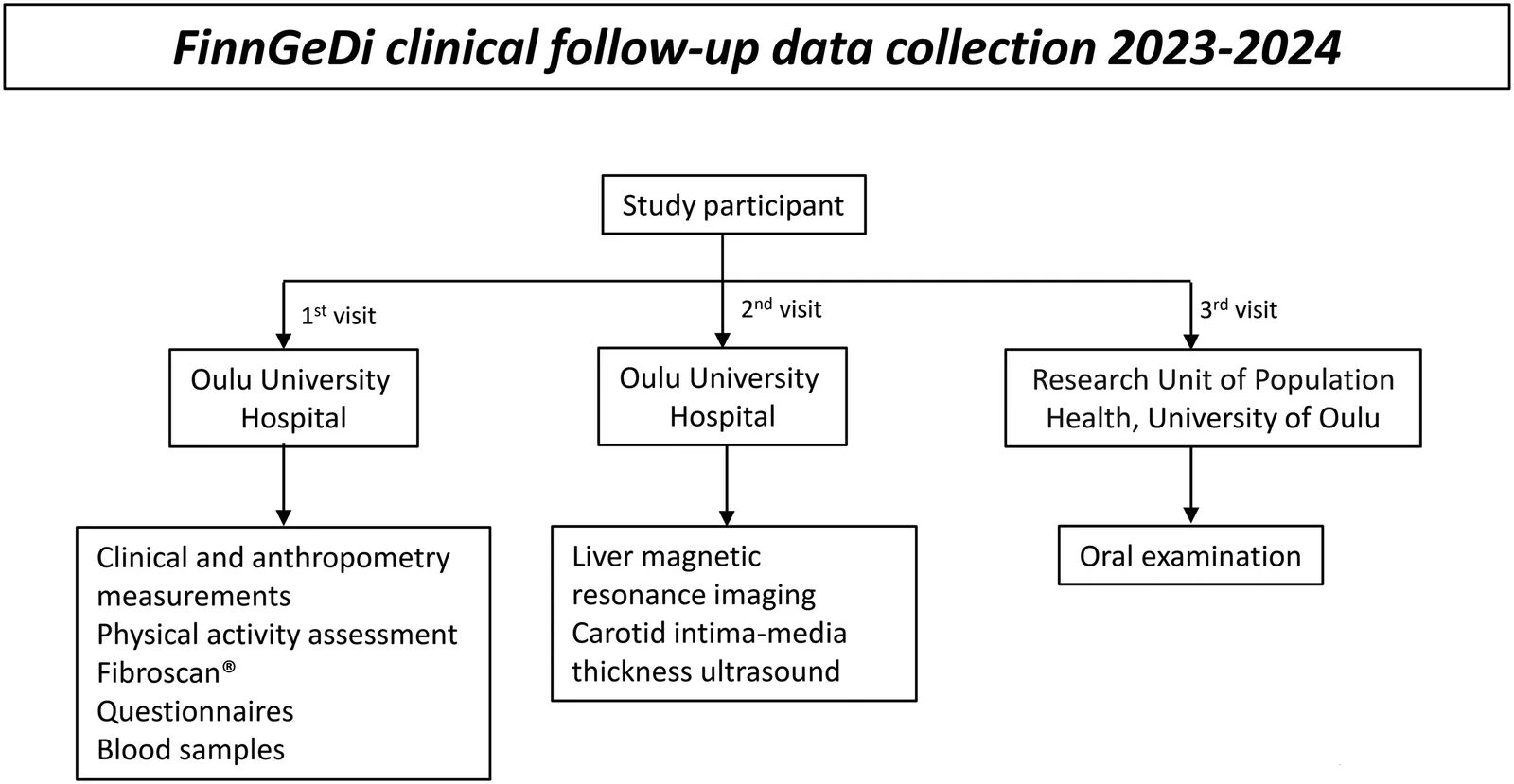
Data-collection process.

A detailed description of the data collected at baseline and at the follow-up is provided in Table 2. The follow-up data collection was carried out during three study visits, as shown in Fig. 2. The participants completed a detailed questionnaire about their educational status, occupation, and menstrual, obstetric, medical, and family history, prior to their visit to the study clinic at Oulu University Hospital, Finland. They spent ∼4 hours at the study clinic, where a series of tests and questionnaires were administered. Anthropometric measurements and body composition to measure muscle and fat mass by bioimpedance (SECA® mBCA 515), and measurement of heart rate variability (HRV) was done using Firstbeat® heart rate monitor for assessing autonomic nervous system function.

**Table 2:** Data collection at baseline (2009–12) and during the follow-up (2023–24).

Measures	Baseline	Follow-up wave
Self-completed questionnaires		
Demographic characteristics		
Age	X	X
Marital status	X	X
Living conditions	X	X
Number of children	X	X
Socio-economic indicators		
Education	X	X
Occupation	X	X
Employment status	X	X
Lifestyle factors		
Smoking	X	X
Alcohol use	X	X
Alcohol Use Disorders Identification Test (AUDIT)		X
Physical activity	X	X
International Physical Activity Questionnaire (IPAQ)		X
Diet	X	X
Oral health questionnaire		
• Tooth-brushing habits		X
• Use of oral health services	X	X
• Presence of oral symptoms	X	X
Menstrual and contraceptive history	X	X
Previous obstetric history	X	X
Medical history	X	X
Family’s medical history	X	X
Sleep and sleep apnoea		
• Pittsburgh Sleep Quality Index (PSQI)		X
• Epworth Sleepiness Scale (ESS)		X
Psychosocial well-being		
• Cognitive functioning [Behaviour Rating Inventory of Executive Function–Adult version (BRIEF-A)]		X
• Self-efficacy [General Self-Efficacy Scale (GSE)]		X
• Adaptive functioning and mental health [Adult Self Report (ASR)]		X
• Health-related quality of life [Short Form 36 Health Survey (SF36)]		X
Clinical examination		
Anthropometry	X	X
Body composition		X
Blood pressure	X	X
Heart-rate variability		X
Physical activity and fitness		
• Triaxial accelerometer measurements (running, cycling, walking, standing, and sitting) for 7 days		X
• Åstrand-Ryhming step test		X
• Push-up test		X
• Hand-grip test		X
Oral health examination		
• Collection of resting and stimulated saliva		X
• Assessment of oral hygiene (plaque)		X
• Caries examination		X
• Periodontal examination (periodontal pocket depth, bleeding on probing, periodontal attachment level)		X
Imaging studies		
• Liver MRI		X
• Vibration-controlled transient elastography (Fibroscan^®^)		X
• Carotid intima media thickness measurement by ultrasound		X
Blood tests		
Routine biochemistry and haematology^a^	X	X
75-g oral glucose tolerance test (OGTT)^b^	X	X
Liver enzymes		X
Inflammatory markers	X	X
Blood for deoxyribonucleic acid (DNA) and ribonucleic acid (RNA) isolation for epigenetic studies	X	X
Faecal and salivary samples for microbiome studies		X

a Routine biochemistry at follow-up included glucose and insulin (fasting and 2-hour), glycated haemoglobin A1c, lipids (total cholesterol, high-density lipoprotein cholesterol, low-density lipoprotein cholesterol, triglycerides, apolipoprotein A1, apolipoprotein B), and thyroid-stimulating hormone. Haematological parameters included haemoglobin, haematocrit, erythrocytes, leukocytes, thrombocytes, mean corpuscular haemoglobin, mean corpuscular haemoglobin concentration, mean corpuscular volume, and red cell distribution width. Liver enzymes included alanine aminotransferase, aspartate aminotransferase, gamma–glutamyl transferase, alkaline phosphatase, and bilirubin. Inflammatory markers included high-sensitivity C-reactive protein, interleukin-6, and tumour necrosis factor.

b OGTT was not performed in the case of previous diabetes mellitus or fasting glucose of >7.0 mmol/l in the follow-up wave.

Venous blood samples were collected and a 2-h 75-g OGTT was carried out. Clinical laboratory measurements included liver enzymes, lipids, inflammatory cytokines, and other markers. Serum and plasma samples were frozen and stored for future analysis. Blood for deoxyribonucleic acid (DNA) and ribonucleic acid (RNA) isolation, to be used in future epigenetic studies, was also collected and stored at −80°C. Faecal samples were collected by using standard collection kits for gut microbiome analyses and stored at −80°C. Transient elastography (Fibroscan^®^) was used to estimate hepatic fibrosis and steatosis.

Standardized and validated questionnaires were administered in the following order. Dietary intake during the previous 12 months was assessed through a semi-quantitative Food Frequency Questionnaire [15], which included 135 food items that are commonly consumed in Finland. Daily intake of energy, nutrients, and food groups was calculated using Fineli^®^, the Finnish Food Composition Database, along with in-house software maintained by the Finnish Institute for Health and Welfare [16]. The final dietary dataset included 80 food groups and 103 nutrients. Physical activity during the past 7 days was assessed using the International Physical Activity Questionnaire (IPAQ) long form [17], which captures occupational, transportation, household, and leisure-time physical activity along with the time spent sitting. Sleep was assessed using the Pittsburgh Sleep Quality Index (PSQI), a 19-item questionnaire measuring subjective sleep quality, sleep latency, sleep duration, habitual sleep efficiency, sleep disturbances, use of sleeping medication, and daytime dysfunction [18]. Daytime sleepiness was evaluated through the Epworth Sleepiness Scale (ESS), a nine-item questionnaire that measures the likelihood of dozing off or falling asleep while engaged in eight everyday situations [19]. Cognitive functioning was assessed via the Behaviour Rating Inventory of Executive Function–Adult version (BRIEF-A) self-report [20], which includes 75 items grouped into nine clinical scales. Self-efficacy was assessed using the ten-item General Self-Efficacy (GSE) scale [21], which measures an individual’s perceived ability to cope with various demands and life challenges. Adaptive social and professional functioning and mental health problems were assessed through the Adult Self Report (ASR) [22], a 126-item questionnaire that includes a wide range of symptoms across multiple domains and also provides diagnostic cut-offs for Diagnostic and Statistical Manual of Mental Disorders (DSM)-oriented scales. The Alcohol Use Disorders Identification Test (AUDIT), a 10-item questionnaire, was used to assess alcohol consumption, drinking behaviours, and alcohol-related problems [23]. Health-related quality of life was measured by using the Short Form 36 Health Survey (SF-36) [24]. All questionnaire data were collected by using REDCap (Research Electronic Data Capture) electronic data-capture tools hosted at the University of Oulu, Finland [25, 26].

Cardiorespiratory fitness was assessed by measuring the heart rate at submaximal endurance (Åstrand-Ryhming step test) [27] in which the participant stepped up and down a level for 4 minutes paced by using a metronome. Muscle fitness and strength were assessed through a 40-second UKK-modified push-up test developed by the Urho Kaleva Kekkonen Institute for Health Promotion Research [28] and grip strength via a hand-grip dynamometer (Saehan). HRV was measured in the resting state, and during and after the 4-minute step test. Physical activity was measured using two Tri-axial AX3 accelerometers (Axivity, Newcastle, UK) attached to the right thigh and lower back for 7 days. A validated machine-learning model was used to identify the time spent in eight physical-activity behaviours: running, cycling, brisk walking, moderate walking, slow walking, standing, sitting, and lying [29, 30].

During the second study visit, quantitative MRI and spectroscopy were performed by using a 1.5-T scanner (Siemens MAGNETOM Sola, software version VA31, Siemens Healthineers, Erlangen, Germany) to assess the fat fraction of the liver through the LiverLab application by Siemens Healthineers. An overview of liver fat fraction was obtained with a Dixon-type sequence and more precise regions of interest were observed by using T2* measurement of tissue and MR spectroscopy to quantify the fat fraction. Ultrasonography for carotid intima media thickness measurement for the assessment of arterial wall thickening was also carried out.

Oral health examinations were carried out at the Research Unit of Population Health at University of Oulu, Finland by the same experienced periodontist for all study participants. At first, resting and stimulated saliva samples were collected for the oral microbiome analyses and the weight, volume, and pH of the saliva were measured. The samples were stored at −80°C for future analyses. The level of oral hygiene was measured by examining visible plaque on buccal sites of the teeth. The cariological status was assessed per tooth surface on all teeth by using the ICDAS criteria [31]. Periodontal examination was carried out on six sites of all teeth by using a manual ball-pointed periodontal probe (LM 8‐520B, Lääkintämuovi, Finland). The following measurements were made: probing crevice/pocket depth, bleeding on probing, and the distance between the gingival margin and the cementoenamel junction. The clinical attachment level was calculated as the distance from the cementoenamel junction to the base of the crevice/pocket. Of the 417 women who participated in the follow-up data collection, 411 (98.6%) completed Fibroscan measurement, 390 (93.5%) underwent liver MRI, 381 (91.4%) completed carotid intima media thickness measurements, and 375 (89.9%) completed the clinical oral examination. Overall, 333 (79.9%) women attended all three clinical visits.

## What has it found? Key findings and publications

Some key characteristics of the study participants are presented in Table 3. The preliminary analysis showed that the metabolic traits in women with prior GDM tend to be more adverse than those in the controls.

**Table 3:** Characteristics of the study participants from the follow-up wave (2023–24).

Parameter	Women with GDM in index pregnancy (*n *= 213)	Control women with no GDM in index pregnancy (*n *= 204)
Age (years)	45.3 (5.1)	43.2 (5.2)
Weight (kg)	79.0 (17.1)	73.7 (15.3)
BMI (kg/m^2^)	29.1 (5.8)	26.9 (5.5)
Waist (cm)	91.7 (13.8)	85.2 (13.1)
Systolic blood pressure (mmHg)	122 (13)	117 (14)
Diastolic blood pressure (mmHg)	85 (9)	81 (9)
Aspartate aminotransferase (U/L)	17.5 (9.4)	17.1 (9.1)
Alanine aminotransferase (U/L)	25.8 (14.1)	22.6 (10.5)
Alkaline phosphatase (U/L)	61.8 (17.8)	56.8 (14.6)
Glutamyl transferase (U/L)	27.7 (26.9)	20.9 (13.4)
Bilirubin (µmol/l)	12.4 (5.2)	13.6 (5.4)
Total cholesterol (mmol/L)	4.9 (0.9)	4.8 (0.7)
High-density lipoprotein cholesterol (mmol/L)	1.4 (0.4)	1.5 (0.3)
Low-density lipoprotein cholesterol (mmol/L)	2.9 (0.7)	2.8 (0.7)
Triglycerides (mmol/L)	1.2 (0.7)	1.0 (0.5)
Fasting glucose (mmol/L)	5.8 (0.8)	5.2 (0.4)
2-h glucose (mmol/L)	6.6 (2.0)	5.5 (1.4)
HbA1c (mmol/mol)	34.5 (4.6)	31.8 (2.6)

Data shown as mean (SD). BMI, body mass index; HbA1c, glycated haemoglobin A1c.

Since the publication of the original cohort profile in 2020, several studies based on the FinnGeDi cohort have been published. These studies have examined various aspects of GDM, including risk factors [32–34], genetic variation [35], maternal and offspring epigenetic studies [36–40], offspring congenital anomalies and mental disorders [41, 42], neonatal outcomes, and oral health associated with GDM [43–45]. These earlier publications highlight the scientific value of the FinnGeDi dataset, providing a strong foundation for the ongoing follow-up analyses presented in this cohort profile update.

At the time of this cohort profile update, no publications based on the follow-up wave (2023–24) have yet been published. Detailed analyses of the collected measures are ongoing and will be reported in future studies.

## What are the main strengths and weaknesses?

The main strengths of the study include a long follow-up period of 11–15 years after the index pregnancy, comprehensive data collection with detailed questionnaires, and face-to-face health assessments, including imaging studies and oral health examinations. Moreover, the assessment of psychosocial well-being, an often-neglected health perspective in long-term studies, through validated questionnaires will provide an overview of the long-term mental health. Another strength is the availability of early-pregnancy laboratory data from the index pregnancy and data from national registers (Supplementary Table 1), which, when combined with the clinical data, will enable a multifaceted assessment of factors influencing GDM-associated health outcomes. One limitation of the study is that the follow-up data collection was conducted at a single study centre due to limited resources, which reduced the number of participants. Of the 964 women invited for follow-up, 417 participated, resulting in a participation rate of 43.3%. When compared with baseline characteristics, the present study participants were slightly older, had lower pre-pregnancy BMI, higher education levels, and were less likely to have smoked during pregnancy than non-participants. This suggests that healthier and more health-conscious women may have been more likely to take part in the follow-up, which should be considered when interpreting the findings.

## Can I get hold of the data? Where can I find out more?

Access restrictions apply to the clinical data, which are is regulated by ethics approvals and individual consents. Access to registry data is subject to permission from the registry authorities. Clinical data and material can be shared with collaborating research projects whenever a joint controller agreement has been made between the University of Oulu and collaborators. For more information, please contact the principal investigator and study coordinator of FinnGeDi, Professor Marja Vääräsmäki, at marja.vaarasmaki@oulu.fi.

## Ethics approval

The study was approved by the regional medical research ethics committee of the Wellbeing services county of North Ostrobothnia (EETTMK: 26/2022) and is carried out according to the principles of the Declaration of Helsinki.

## Supplementary Material

dyag101_Supplementary_Data

## Data Availability

See ‘Can I get hold of the data?’, above.
