# Supplementary material for: Cohort Profile Update: The Finnish Gestational Diabetes (FinnGeDi) study
Source: Int J Epidemiol. 2026 Jul 9;55(4):dyag101. doi: 10.1093/ije/dyag101 (PMC13348704; doi:10.1093/ije/dyag101)
Supplement: dyag101_Supplementary_Data [file dyag101_supplementary_data.zip › ije-2025-09-1807-File006.docx]

**Cohort Profile Update: The Finnish Gestational Diabetes (FinnGeDi) Study**

**Supplementary Table S1. Register data**

| **Institution** | **Register** | **Data** | **Time** |
| --- | --- | --- | --- |
| Finnish Institute of Health and Welfare | Medical Birth Register | Pregnancy and neonatal data | 1987 – 2022 |
|  | Hospital discharge diagnoses | Diagnoses*  Hospitalization* | 1987 – 2022 |
| Digital and Population Data Services Agency | Population information system | Residential coordinates, language, marriage status | 1960 – 2023 |
| Social Insurance Institution of Finland | Reimbursement of drugs | Special reimbursement codes* | 1964 – 2022 |
|  |  | Purchase of medicine* | 1993 – 2022 |
|  | Prescription centre and archive | Electronic prescriptions* | 2010 – 2022 |

*Data selected to emphasize metabolic, cardiovascular and mental health.
