# Supplementary material for: Cohort Profile Update: The Finnish Gestational Diabetes (FinnGeDi) study
Source: Int J Epidemiol. 2026 Jul 9;55(4):dyag101. doi: 10.1093/ije/dyag101 (PMC13348704; doi:10.1093/ije/dyag101)
Supplement: dyag101_Supplementary_Data [file dyag101_supplementary_data.zip › ije-2025-09-1807-File005.docx]

**Cohort Profile Update: The Finnish Gestational Diabetes (FinnGeDi) Study**

**Author contribution statement**

Shilpa Lingaiah: Overall study design, Data collection implementation in REDCap, Formal analysis, Writing–original draft.

Elina Keikkala: Retrieval of contact information from the Digital and Population Data Services Agency (DVV), Formal analysis, Writing–Review and editing.

Sanna Mustaniemi: Writing–Review and editing.

Pekka Ylöstalo: Provided expertise in oral health study design and contributed to oral data collection, Writing–Review and editing.

Tellervo Tervonen: Provided expertise in oral health study design and contributed to oral data collection, Writing–Review and editing.

Marja-Liisa Laitala: Contributed to oral data collection, Writing–Review and editing.

Hilkka Pernu: Performed clinical oral examinations of all participants, Writing–Review and editing.

Eveliina Lammentausta: MRI data collection, Writing–Review and editing.

Mika Nevalainen: Provided consultation on MRI examinations, Writing–Review and editing.

Jyri-Johan Paakki: Provided consultation on ultrasound examinations, Writing–Review and editing.

Satu Männistö: Development and expertise in food frequency questionnaires (FFQ), Writing–Review and editing.

Niina Kaartinen: Provided expertise in food frequency questionnaires (FFQ), Writing–Review and editing.

Marjaana Tikanmäki: Provided expertise in physical activity assessment design. Writing–Review and editing.

Annukka Torkki: Writing–Review and editing.

Fredrik Åberg: Contributed expert input on MASLD-related aspects of the study, Writing–Review and editing.

Anneli Pouta: Member of the core team for the baseline FinnGeDi study, Writing–Review and editing.

Risto Kaaja: Member of the core team for the baseline FinnGeDi study, Writing–Review and editing.

Johan G Eriksson: Member of the core team for the baseline FinnGeDi study, Writing–Review and editing.

Hannele Laivuori: Member of the core team for the baseline FinnGeDi study, Writing–Review and editing.

Mika Gissler: Member of the core team for the baseline FinnGeDi study, Writing–Review and editing.

Tuija Männistö: Provided expertise in laboratory medicine, Writing–Review and editing.

Pirkko Pussinen: Provided expertise in microbiome study design, Writing–Review and editing.

Terhi Ruuska-Loewald: Provided expertise in microbiome study design, Writing–Review and editing.

Kari Anne Indredavik Evensen: Provided expertise in physical activity assessment design, Writing–Review and editing.

Mikko Tulppo: Provided expertise in HRV study design, Writing–Review and editing.

Julia Jäkel: Provided expertise in mental wellbeing study design, Writing–Review and editing

Eero Kajantie: FinnGeDi baseline study group, Resources, Writing–Review and editing

Marja Vääräsmäki: Overall study design, Resources, Writing–Review and editing, Supervision, Funding acquisition.

All authors approved the final version.
